# Application of precise neutron focusing mirrors for neutron reflectometry: latest results and future prospects

**DOI:** 10.1107/S1600576720013059

**Published:** 2020-10-26

**Authors:** Norifumi L. Yamada, Takuya Hosobata, Fumiya Nemoto, Koichiro Hori, Masahiro Hino, Jun Izumi, Kota Suzuki, Masaaki Hirayama, Ryoji Kanno, Yutaka Yamagata

**Affiliations:** a Institute of Materials Structure Science, High Energy Accelerator Research Organization, Tokai, Naka, Ibaraki 319-1106, Japan; b Materials and Life Science Experimental Facility, Japan Proton Accelerator Research Complex, Tokai, Naka, Ibaraki 319-1195, Japan; c RIKEN Center for Advanced Photonics, RIKEN, Wako, Saitama 351-0198, Japan; d Department of Materials Science and Engineering, National Defense Academy, Yokosuka, Kanagawa 239-8686, Japan; e Sumitomo Rubber Industries Ltd, Kobe, Hyogo 651-0071, Japan; f Kyoto University Institute for Integrated Radiation and Nuclear Science, Kumatori, Osaka 590-0494, Japan; g Interdisciplinary Graduate School of Science and Engineering, Tokyo Institute of Technology, Yokohama, Kanagawa 226-8502, Japan; h All-Solid-State Battery Unit, Institute of Innovation Research, Tokyo Institute of Technology, Yokohama, Kanagawa 226-8503, Japan

**Keywords:** neutron reflectometry, focusing mirrors, Li-ion batteries

## Abstract

Neutron reflectometry (NR) is a powerful tool for providing insight into the evolution of interfacial structures, for example via *operando* measurements for electrode–electrolyte interfaces, with a spatial resolution of nanometres. The time resolution of NR, which ranges from seconds to minutes depending on the reflection intensity, unfortunately remains low, particularly for small samples made of state-of-the-art materials even with the latest neutron reflectometers. To overcome this problem, a large-area focusing supermirror manufactured with ultra-precision machining has been employed to enhance the neutron flux at the sample, and a gain of approximately 100% in the neutron flux was achieved. Using this mirror, a reflectivity measurement was performed on a thin cathode film on an SrTiO_3_ substrate in contact with an electrolyte with a small area of 15 × 15 mm. The reflectivity data obtained with the focusing mirror were consistent with those without the mirror, but the acquisition time was shortened to half that of the original, which is an important milestone for rapid measurements with a limited reciprocal space. Furthermore, a method for further upgrades that will reveal the structural evolution with a wide reciprocal space is proposed, by applying this mirror for multi-incident-angle neutron reflectometry.

## Abstract 2

A large-area focusing supermirror manufactured with ultra-precision machining has been employed at the SOFIA reflectometer at the J-PARC Materials and Life Science Experimental Facility, and a gain of approximately 100% in the neutron flux was achieved. For future upgrade, optics using the focusing mirror for multi-incident-angle neutron reflectometry are proposed, in order to reveal evolutions of interfacial structures for *operando* measurements with a wide reciprocal space.

## 1. Introduction   

Neutron reflectometry (NR) is a powerful tool for observing surface and interfacial structures along the normal direction to a substrate in the spatial range from nanometres to hundreds of nanometres. As neutrons are sensitive to light elements, NR enables the evaluation of the depth distribution of the elemental composition in energy materials containing hydrogen and lithium. Moreover, *in situ* observations of thin films on solids immersed in liquid are widely conducted because neutrons can illuminate the film through the substrate owing to the high transmissibility. Furthermore, semi-white neutrons can be used by applying a time-of-flight (TOF) method with pulsed neutrons, as the wavelength of the neutrons, λ, is dependent on the velocity based on the TOF from the neutron generation to the detection. This feature enables the measurement of the reflectivity over a wide reciprocal space without incident angle scanning. For example, the momentum transfer vector **Q** is normal to the reflection plane, and its magnitude *Q*_*z*_ is 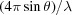, where the incident angle θ is the same as the reflection angle under specular reflection conditions. By using semi-white neutrons one can cover a wide *Q*_*z*_ range by scanning λ at a fixed θ, whereas only one *Q* *_z_* point can be measured by the use of monochromatic neutrons. On the basis of the above advantages, NR has been applied extensively for investigating interfacial structures, not only for the purpose of basic interfacial science but also in functional materials. For example, the electrodes of lithium ion batteries (LIBs) are a key target for NR because they are composed of a complex of Li compounds and organic binders containing light elements that are sensitive to neutrons, and the interface with a liquid electrolyte where electrochemical reactions occur is at the frontline of research. More specifically, interfacial layers formed at electrode–electrolyte interfaces and the distributions of Li ions not only under constant voltages (Hirayama, Yonemura *et al.*, 2010 ▸; Owejan *et al.*, 2012 ▸; Browning *et al.*, 2014 ▸; Minato *et al.*, 2016 ▸; Browning *et al.*, 2019 ▸) but also in *operando* studies during charging/discharging processes (Kawaura *et al.*, 2016 ▸; Kawaura *et al.*, 2020 ▸) have been investigated using NR to date.

Several technical limitations exist in expanding the use of time-slicing NR for studies of kinetics of nanoscale interfacial structures, such as *operando* measurements: most notably, the time resolution is limited by the statistics of reflected neutrons, and the observable *Q*_*z*_ range is limited by the wavelength band of the incident neutrons. For a sample having a typical size for NR at present, namely several inches in diameter, one NR profile at a small θ can be obtained within several seconds to minutes, depending on the neutron flux of the instrument and the reflectivity of the sample, where the reflectivity typically decreases from unity (total reflection) to 10^−3^–10^−4^. In this case, the ratio of the maximum to minimum *Q*_*z*_ values covered by one incident angle is the same as the ratio of the maximum to minimum values of the available wavelength. This is mainly defined by the most efficient wavelength providing the highest intensity at the Maxwell peak, and the TOF length and repetition rate of the neutron pulses limiting the slowest neutrons: that is, the longest wavelength. This ratio limits the width of the *Q*_*z*_ range at a given incident angle for time-slicing measurements, although reflectivity data with a wider *Q*_*z*_ region are required for taking into account the interference arising from a wider scale for more accurate data analysis, particularly for complex multi-layered structures. The expansion of the wavelength band and/or merging of the data sets obtained at different θ values are simple solutions for extending the *Q*_*z*_ region. The former method can be performed by reducing the repetition rate; however, additional time is required to obtain the same number of neutron pulses for maintaining the statistics. In contrast, the latter approach requires a loss of time to change the optics, incident angle and detector angle. Furthermore, additional accumulation time is required for obtaining data at a higher *Q*_*z*_, as the reflectivity decreases drastically with an increase in *Q*_*z*_; it is proportional to 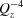 when the surface roughness is negligibly low for the *Q*_*z*_ range and decreases more steeply when the *Q*_*z*_ value is sufficiently high to detect the roughness effect. Furthermore, smaller samples are preferred to provide high quality, especially for state-of-the-art-materials, which leads to a decrease in the reflection intensity. The focusing optics offer a means of overcoming this problem. In this study, we concentrate on planar elliptic mirrors as a focusing device because they can focus semi-white neutrons on a small beam spot with large beam divergence.

**Figure 1:**
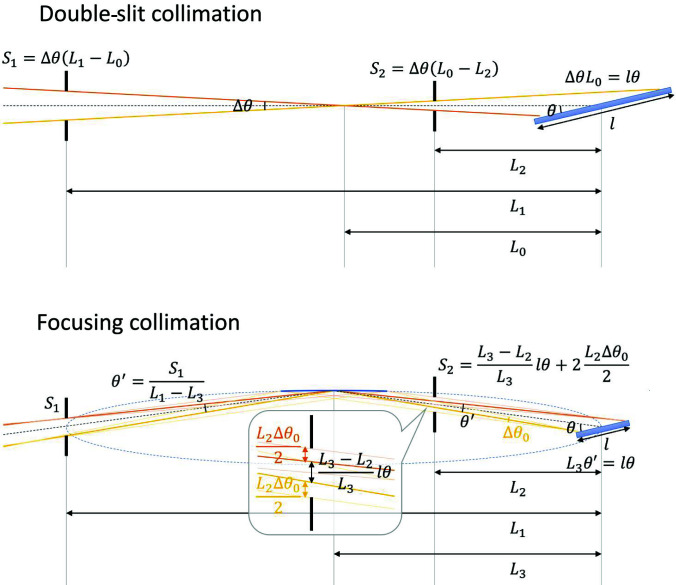
Comparison of conventional double-slit collimation and collimation with a focusing mirror. Whereas a beam with a low divergence is required to illuminate a small sample with conventional collimation, a beam with a large divergence can be employed on a small sample with focusing collimation.

Fig. 1 ▸ presents rough sketches of the beam optics applied for NR in this study: the upper sketch indicates the conventional optics with double slits, while the other represents optics with a focusing mirror (see supporting information for detail). In the former case, the widths of the first slit *S*_1_ and second slit *S*_2_ depend on the sample size *l*, incident angle θ, beam divergence Δθ, and distances from the sample to the first slit *L*_1_ and to the second slit *L*_2_, as follows:
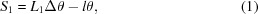


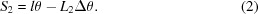
As the product of the slit apertures, *S*_1_ *S*_2_, is proportional to the beam flux, the Δθ value yielding the highest beam flux, Δθ_0_, can be described as follows:
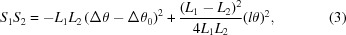


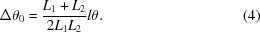
This means that a smaller divergence is required for smaller samples. Although a larger Δθ_0_ (that is, a higher beam flux) can be achieved by reducing the *L*_2_ value, this causes a limitation in the sample environment owing to the interruption by the slit near the sample. However, the beam size at the sample position can be controlled by only *S*_1_, and the divergence is independent of the beam divergence for the focusing optics, as follows:
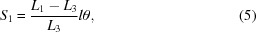


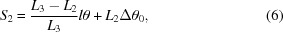
where *L*_3_ is the distance from the sample to the focusing mirror and Δθ_0_ is determined by the solid angle of the focusing mirror from the sample position: that is, it is independent of the sample size (*S*_2_ is simply used for background reduction by omitting neutrons that are not reflected by the mirror and is inessential for creating the optics). Thus, the focusing optics can introduce a high-flux neutron beam with large beam divergence even on a small limited area and can be free from interruption by the slit near the sample.

Focusing optics with a Montel mirror system consisting of four supermirrors that have a planar elliptic shape, known as a Selene guide, have already been realized and are in use in the Amor reflectometer in the SINQ neutron source at the Paul Scherrer Institute (Stahn & Glavic, 2016 ▸). In these optics, neutrons spreading from a virtual source are converged on a midpoint between the source and sample, the separation of which is 4000 mm, by a pair of the mirrors focusing in the vertical and horizontal directions, respectively, and are converged on the sample by another pair of mirrors (as the length of the mirrors, namely 1200 mm, is so long that the coma aberration effect is non-negligible, two sets of focusing mirrors were introduced to correct the aberration). The optics operate effectively and can typically converge the neutrons down to 0.8 mm, and to approximately half this value with less beam divergence by cutting the neutrons that originate from the figure error of the mirrors with a slit (Stahn, 2019 ▸). This minimum beam size is sufficiently small to measure the total reflection at the smallest incident angle for typical samples of several inches in diameter: the illuminated length *l* for θ = 1.1° is elongated to 42 mm 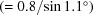. However, the sample size for state-of-the-art materials is substantially smaller: typically approximately 1 cm. Moreover, the intensity gain achieved by applying focusing optics is more enhanced for smaller samples. Hence, a smaller beam spot is highly desirable when using focusing optics for such samples.

On the basis of the background presented above, we have been developing focusing mirrors to enhance the neutron flux at small samples. Firstly, we attempted to realize the elliptical shape by bending a large mirror on a glass substrate (Torikai *et al.*, 2011 ▸; Morita *et al.*, 2016 ▸), and we manufactured a metal substrate with an elliptical shape by means of ultra-precision machining (Takeda *et al.*, 2016 ▸; Hosobata *et al.*, 2017 ▸, 2019 ▸). Finally, we succeeded in fabricating a sufficiently precise focusing mirror with the metal substrate to be utilized as the focusing optics for NR: the dimensions were 550 mm in length and 60 mm in width. The semi-major and -minor axes of the planar elliptic shape measured by an optical interferometer (Verifier QPZ, Zygo Corporation) were 2219 and 21.5 mm, respectively. The mirror slope error was 27.7 µrad, and the incident angle of the neutrons evaluated by the shape was 9.7 mrad. The critical angle was 2.93 times greater than that of an Ni mirror, that is the ‘*m* value’ is *m* = 2.93. The reflectivity at the critical angle was 86.2 ± 2.5% over the entire area (Hosobata *et al.*, 2019 ▸). In this study, we provide a detailed evaluation of the focusing optics with the latest mirror, a comparison with the conventional optics, and the reflectivity data of a small LIB sample with and without the focusing mirror. Furthermore, we propose a concept for extending the *Q*_*z*_ region by taking advantage of the more flexible optics offered by focusing mirrors compared with conventional double slits.

## 2. Methods and results   

### 2.1. Evaluation of focusing optics   

The experiment was performed using the SOFIA reflectometer with a pulsed neutron source at the Materials and Life Science Experimental Facility (MLF) in the Japan Proton Accelerator Research Complex (J-PARC MLF) (Yamada *et al.*, 2011 ▸; Mitamura *et al.*, 2013 ▸), for which *L*_1_ = 4300 mm, *L*_2_ = 200 mm and *L*_3_ = 2150 mm. For the mirror alignment, tilt and height scans were performed in the direct beam geometry to determine the tilt and height that caused the mirror surface to be parallel to and at the center of the beam, respectively. Thereafter, the mirror tilt angle was scanned while maintaining the height to provide the smallest beam width at the sample position in the focal plane. A slit with an aperture of 5 µm made of Cd (see supporting information for details) was placed at the sample position and scanned to measure the intensity profiles because the beam size was too small to resolve with a position-sensitive detector. Finally, the optimal incident angle for the focusing mirror was evaluated as 0.5395°, which was slightly smaller than the value of 9.7 mrad (= 0.56°) that was evaluated from the mirror shape.

**Figure 2:**
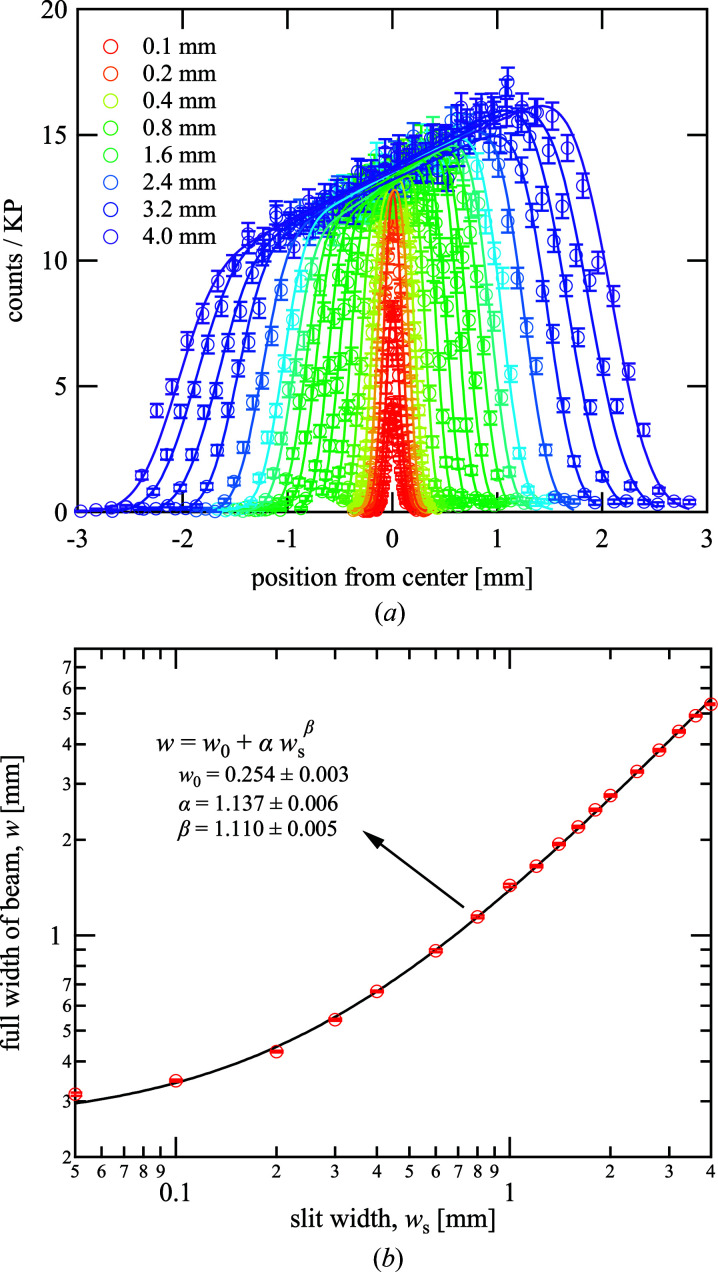
(*a*) Neutron intensity profiles and (*b*) full width of beam at sample position depending on slit aperture, in which both the sample and slit positions are at the focal points of the focusing mirror. The symbols indicate the values at each point, the error bars represent the statistical error and the solid lines are the fitting results.

Fig. 2 ▸(*a*) presents the neutron intensity profile at the sample position in the focal plane when changing the slit aperture from 0.05 to 4.0 mm at the virtual source in the other focal plane. Whereas the peak intensity of the profile increased with the slit opening when the aperture was small, the intensity increase was suddenly suppressed when the aperture was more than 0.2 mm, and the beam width increased with the aperture instead. In this case, a homogeneous beam source in the focal plane ideally formed an image of an isosceles trapezoid in the other focal plane, taking the aberration into account, which was consistent with the profile at the large aperture, except for the slope on the top originating from the intensity distribution at the neutron source. However, the profile at the small aperture appeared as a Gaussian shape because the slight slope error of the focusing mirror, which was approximately tens of microradians, caused the image to be blurred. Next, we performed fitting of the beam profiles to evaluate the beam width at the sample position quantitatively.

For small apertures (≤0.2 mm), the profiles were fitted with Gaussian functions, whereas the following function was applied for larger apertures:
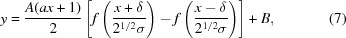


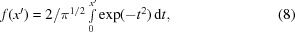
where *y* is the beam intensity at the position from the beam center *x*, *A* is the intensity at the center (*x* = 0), *a* is the slope at the top originating from the beam inhomogeneity, δ is the distance from the beam center to the midpoint of the trapezoid leg, expressed as the error function *f*(*x*′), σ is the standard deviation of the error function and *B* is the background. Fig. 2 ▸(*b*) presents the dependence of the full width of the beam *w*, determined as 2(2σ + δ), on the slit aperture *w*_s_, where δ = 0 for the data fitted with the Gaussian function. The minimum beam width was approximately 0.3 mm at *w*_s_ = 0.05 mm, which was less than half of that achieved by the Selene guide. The increase in *w* was slow when *w*_s_ was small and the slope gradually increased with *w*_s_. The fitting to *w* with respect to *w*_s_ was performed with the power function
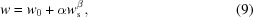
and is used for adjusting *w*_s_ to be the desired value of *w* of more than *w* = 0.3 mm henceforth.

### 2.2. Comparison with conventional optics   

Next, we performed *in situ* measurements of an LIB with the focusing optics and compared the results with those of the conventional slit optics. An electrode comprising an epitaxial thin film of LiCoO_2_ on an epitaxial thin film of SrRuO_3_ was fabricated on an Nb-doped (0.2%) single-crystal SrTiO_3_ (100) substrate with dimensions of 20 mm (width) × 20 mm (depth) × 5 mm (thickness) using a KrF excimer laser with a wavelength of 248 nm and a pulsed laser deposition apparatus (PLAD131, AOV Inc.). As LiCoO_2_ cathodes modified by very thin oxide layers with a thickness of several nanometres have been widely established to exhibit superior cycle stability and high rate capability compared with unmodified LiCoO_2_ (Hirayama, Sonoyama, Ito *et al.*, 2007 ▸; Hirayama, Sonoyama, Abe *et al.*, 2007 ▸; Hirayama, Ido *et al.*, 2010 ▸), direct observation of the electrochemical interface during battery operation is the key to elucidating the modification mechanism by the oxide layer. This cathode was packed with a custom-made electrochemical cell (Yonemura *et al.*, 2014 ▸) and soaked in an electrolyte LiPF_6_ solution consisting of a mixture of deuterated ethylene carbonate and deuterated diethyl carbonate, with a counter-electrode made of Li metal in an Ar-substituted glove box. For the pre-conditioning, charge and discharge cycles were repeated three times prior to the measurement, and the voltage was maintained at the open-circuit voltage of the sample, namely 3.9 V, during the measurement. The neutrons were introduced from the substrate side, and an area of 15 × 15 mm of the electrode in contact with the electrolyte was illuminated. The ratio of the reflection intensity to the intensity of the direct beam through the substrate was evaluated as the reflectivity, as illustrated in Fig. 5 (Section 2.3).

**Figure 3:**
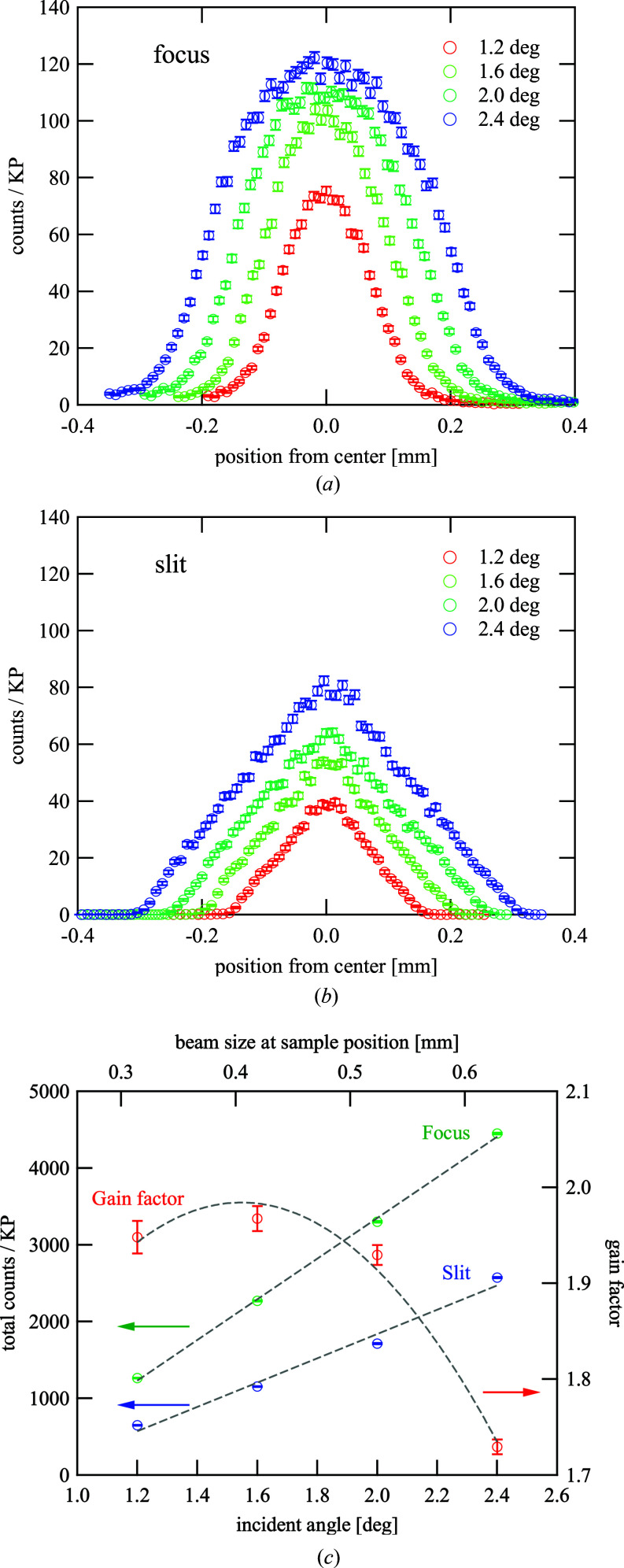
Comparison of beam profiles between focusing optics and slit optics. (*a*), (*b*) The intensity profiles for the focusing and conventional slit optics, respectively. (*c*) Total intensity depending on incident angle θ for both optics and the gain factor evaluated with total intensity.

The intensity profiles at the sample position with the focusing optics and slit optics were compared prior to the NR experiment. Figs. 3 ▸(*a*) and 3 ▸(*b*) present the profiles when changing the slit apertures according to equations (2)–(6) for illuminating the area of 15 × 15 mm with neutrons of different incident angles θ using the focusing and slit optics, respectively. For the focusing optics, the profile exhibited a Gaussian-like shape at θ = 1.2°, where the beam size was almost the minimum size achievable by the focusing optics, and it gradually changed into a trapezoid-like shape with increasing θ, as noted previously. However, all of the profiles for the slit optics exhibited triangular shapes, which could be observed when the beam intensity was optimized. Note that the slope of the triangle creating the peak was more gradual than that for the focusing optics, which made a difference in the beam flux. However, as the increase in the peak intensity for the focusing optics was suppressed when the peak became a trapezoid-like shape with increasing θ, the ratio of the total count with the focusing mirror to that without the mirror (gain factor) exhibited a peak at around θ = 1.6°, as illustrated in Fig. 3 ▸. Therefore, we selected θ = 1.6° to compare the reflectivity data between the two optics.

### 2.3. *In situ* NR experiment   

**Figure 4:**
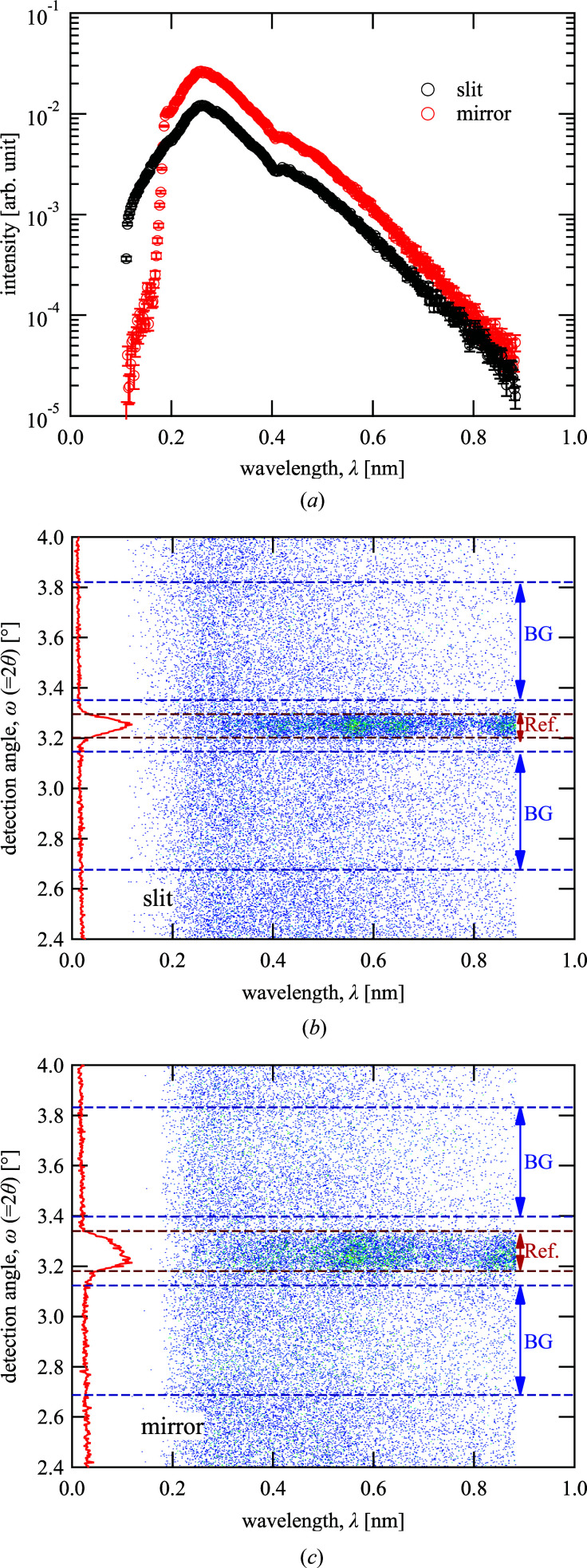
Neutron intensity distribution of direct beam and reflected beam. (*a*) Intensity profile of the direct beam through the substrate as a function of wavelength, and the intensity maps of the reflected beam as a function of wavelength and detection angle for (*b*) double-slit optics and (*c*) focusing optics.

Fig. 4 ▸(*a*) presents the neutron intensity of the direct beam through the substrate depending on the neutron wavelength λ, based on the TOF method. Although the intensity with the focusing optics was more than that with the double slits for λ > 0.19 nm, a sudden decrease in the intensity occurred around λ = 0.19 nm in the case of the focusing optics and the relative intensity was inverted for λ < 0.19 nm. The sudden drop originated from the cut-off of the supermirror reflection, which could transport neutrons with λ more than the critical value determined by the incident angle to the mirror, 0.5395°, and the *m* value of the mirror, 2.93. Better statistics can normally be achieved from longer λ for NR with pulsed neutrons, because the reflectivity increases drastically with λ except in the total reflection region. This means that the cut-off due to the supermirror had a small effect on the NR because the use of neutrons with shorter λ than that at the Maxwell peak was inefficient. Figs. 4 ▸(*b*) and 4 ▸(*c*) present the intensity maps depending on λ and the detection angle from the direct beam position ω, in which ω was converted from the neutron detection position on the area detector with an angular resolution of approximately 0.02°. For specular reflection, ω is equal to 2θ as the reflection angle was the same as the incident angle θ. In fact, signals of reflection could be observed at around ω = 3.2° in both cases. The distribution of the reflection in ω for the focusing optics was wider than that for the double slits, as larger beam divergence was accepted for the focusing optics. This was the origin of the enhancement in the beam flux, and the accumulation time to obtain 20 000 counts was reduced by a factor of 2.16 times: 258 000 neutron pulses (equivalent to 10 300 s) were required for the double slits, whereas 119 000 (equivalent to 4760 s) were required for the focusing optics.

The conversion from the reflection intensity to the reflectivity as a function of the momentum transfer normal to the substrate, *Q*_*z*_, was performed, taking into account the λ dependence of the incident beam intensity indicated in Fig. 4 ▸(*a*), and the correction of *Q*_*z*_ depending on λ and ω (= 2θ) in the intensity maps presented in Figs. 4 ▸(*b*) and 4 ▸(*c*), as described in the literature (Cubitt *et al.*, 2015 ▸). By means of this treatment, we could evaluate the reflectivity profiles with the same *Q*_*z*_ resolution, even when the beam divergences of the data were different, if the ω resolution was sufficiently small. In the conversion, the region for the reflectivity evaluation was limited to the center region with the signals of the specular reflection, and that for the background evaluation was limited to the surrounding region with no reflection signal; these regions are denoted as ‘Ref.’ and ‘BG’ in the figures, respectively. The background signal as a function of λ was evaluated under the assumption that the background was dependent only on λ, and the background signal in the reflection region was subtracted in the conversion.

**Figure 5:**
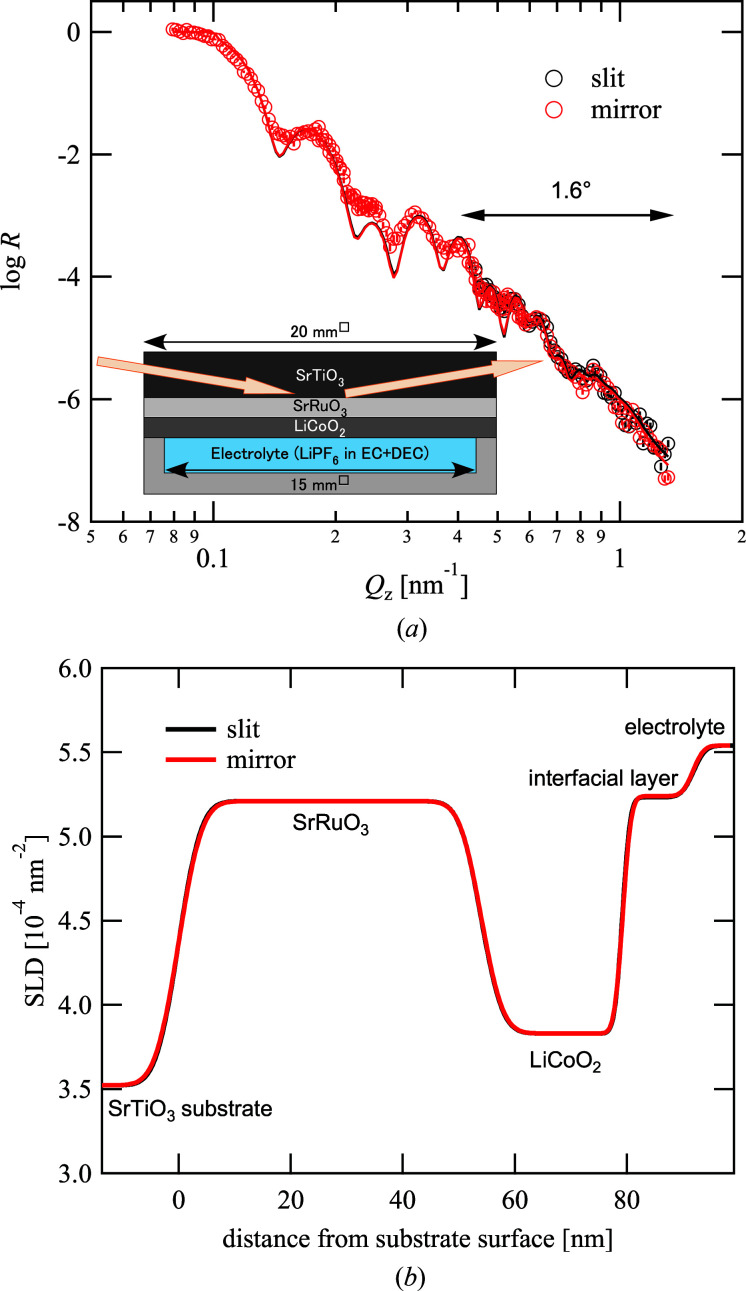
Reflectivity profile and evaluated scattering length density profile of model electrode. (*a*) Reflectivity profiles obtained with double slits and focusing optics (symbols), and fitting results (solid curves). As the focusing optics cannot be applied for low incident angles owing to the limitation of the beam size, the reflectivity data at low *Q*_*z*_ values are shared for the two data points. (*b*) Scattering length density of electrode evaluated by fitting reflectivity data.

Fig. 5 ▸(*a*) presents the reflectivity profiles with the two optics at θ = 1.6°. As the focusing optics could not be applied for lower angles to cover the low-*Q*_*z*_ region, θ = 0.3 and 0.7°, owing to the limitation of the beam size, the data at the lower angles obtained with the double-slit optics were used for both data sets at θ = 1.6°, to be merged into one set of reflectivity data. The two data points at θ = 1.6° were consistent with one another even though a slight discrepancy existed. To evaluate the effect of the slight discrepancy on the data analysis, the two data sets were analyzed by least-squares fitting with the reflectivity based on the Parratt recursive formula (Parratt, 1954 ▸) using the *Motofit* software (Nelson, 2006 ▸). The profiles could be fitted effectively, except for the fringes around 0.25 nm^−1^, with the model taking into account an interfacial layer on the electrode, in which the scattering length densities (SLDs) of the substrate materials and electrode were fixed to be the same as those of the bulk materials. The obtained fitting parameters presented in Table 1 ▸ and the SLD profiles along the direction normal to the substrate indicated in Fig. 5 ▸(*b*) were consistent with one another. Hence, we could conclude that the interfacial layer related to the electrochemical reaction on the cathode was successfully observed with and without the focusing mirror, and the optics worked effectively to reduce the exposure time.

## 3. Future prospects   

By employing the focusing mirror, the enhancement of the beam flux demonstrated above can realize improved time resolution for time-slicing NR measurements. However, the limitation of the *Q*_*z*_ range remains to be solved to achieve more reliable data analysis in a wider reciprocal space. Therefore, we propose the concept of ‘multi-incident-angle neutron reflectometry’ (MI–NR) with focusing mirror optics.

The concept of MI–NR has been put forward by several researchers independently and different methods have been proposed for its realization. For example, C. F. Majkrzak proposed the CANDOR reflectometer at the NIST Center for Neutron Research, in which continuous white neutrons are introduced by converging collimators on a sample, and reflection beams passing through secondary collimators placed at the specular reflection positions are counted by analyzer and detector pairs operating as energy-dispersive detectors (Majkrzak *et al.*, 2020 ▸). Another example is the FREIA reflectometer proposed by H. Wacklin at the European Spallation Source. In this setup, a sample is illuminated by three pulsed beams independently collimated by three pairs of double slits with discrete incident angles, and the neutrons reflected at the sample surface are independently counted by detectors (Wacklin, 2016 ▸). The essential point of these concepts is that neutrons introduced at different incidence angles can be detected separately at different positions because the reflection angle is normally the same as the incidence angle. For both cases, the reflectometers are being constructed from scratch and the beam transport system can be designed to match the optics to realize MI–NR. By applying focusing mirrors, the optics for MI–NR can be achieved without modifications of the transport system, by taking advantage of the flexibility for designing optics.

**Figure 6:**
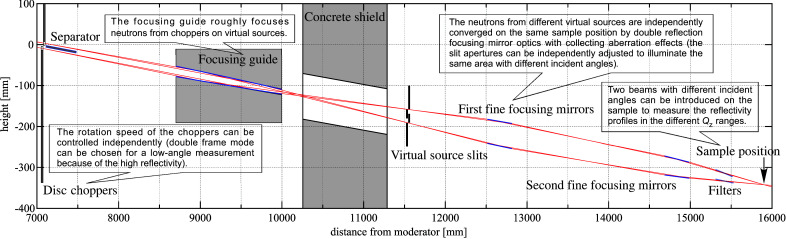
Rough design of MI–NR optics for the SOFIA reflectometer with focusing mirrors.

Fig. 6 ▸ presents a rough design of the optics for realizing MI–NR at the SOFIA reflectometer with focusing mirrors. The neutrons at the exit of the guide tube are separated into up and down parts and independently chopped by disc choppers for controlling the repetition rate (25 or 12.5 Hz). The two beams are roughly focused on different virtual sources 4500 mm from the choppers: one is for a lower incident angle and the other is for a higher one. As a shorter exposure time is generally required for a lower angle, the repetition rate can be reduced to extend the wavelength band in exchange for less flux. The aperture sizes of the virtual sources can be controlled independently by the slits according to equation (6), and the images are formed at the sample position 4300 mm from the sources. In this case, pairs of focusing mirrors to reflect the neutrons twice are employed for realizing images less than 0.3 mm, because the effect of the slope error may be halved by reducing the focal length to half. Furthermore, the effect of aberrations owing to coma and gravity can be corrected by the double reflection as in the Selene guide. The neutrons reflected by the focusing mirrors pass through frame-overlap mirrors, cutting the slow neutrons, and finally, the sample is illuminated by neutrons with two different incident angles.

Table 2 ▸ presents the design parameters for realizing the MI–NR optics with focusing mirrors on the SOFIA reflectometer. The values are moderate and realistic; for example, the critical angle of the fine-focusing mirror is three times greater than that of an Ni mirror, at *Q*_c_ = 0.217 nm^−1^, which is almost the same as that of the focusing mirror we installed in this study. All of the optical components are placed to be symmetric about the original beam center, and the difference between the beam tilts at the sample position with and without the optics is 1.16°. This results in downward beams with tilt angles of 1.04 and 3.36° because the original tilt angle without the mirror is 2.22°. The achievable *Q*_*z*_ ranges with these tilt angles are summarized in Table 3 ▸. For free interfaces, such as air–liquid and liquid–liquid interfaces, the sample surface cannot be tilted by a goniometer. In this case, *Q*_*z*_ ranges from 0.13 nm^−1^, yielding the total reflection of heavy water (*Q*_*z*_ < 0.18 nm^−1^), to 2.95 nm^−1^, with the most efficient wavelength at the Maxwell peak (0.25 nm) yielding the highest intensity. This is greater than the typical upper limit achieved by NR at a *Q*_*z*_ of approximately 2.5 nm^−1^. This means that the *Q*_*z*_ range for typical samples can be covered, even without any angle scan. For interfaces with a solid phase, such as electrode–electrolyte interfaces, the minimum *Q*_*z*_ required for NR is typically 0.1 nm^−1^ as the total reflection of Si, which is used commonly as a substrate, occurs at *Q*_*z*_ < 0.1 nm^−1^. In this case, the sample surface can be tilted and we can access a lower *Q*_*z*_ region by decreasing the incident angle, whereas the *Q*_*z*_ ranges covered by the lower and higher angles become less overlapped. Again, it should be noted that a shorter exposure time is required for obtaining data of sufficient statistics at the lower angle owing to the greater reflection intensity. Subsequently, effective statistics can be achieved even with a less efficient wavelength at λ < 0.25 nm for the lower angle until the data obtained for the higher angle are finished. This enables us to create a strong overlap in the *Q*_*z*_ region between the two angles and to measure the entire reflectivity profile without any angle scan from the total reflection region up to more than 2.5 nm^−1^, which is the typical upper limit for NR, for solid samples as well.

Note here that the optics of MI–NR can reduce the minimum beam size at the sample, which comes from the slope error of the mirror as mentioned before. As the broadening of the mirror is evaluated with the product of the slope error and focal length, the beam size is halved for a mirror with half the focal length even with the same slope error. Although it is broadened again by the second mirror to be 2^1/2^ times more, that is, the minimum size will be 0.3/2 × 2^1/2^ = 0.21 mm, we still have room to improve the slope error because the smaller mirror size for MI–NR makes machining easier. If the minimum beam size is less than 0.19 mm, MI–NR can be applied for a sample even with a size of 15 × 15 mm. In contrast, the effect of gravity is expected to affect the beam size, especially for slow neutrons such as λ = 1.77 nm (see supporting information for details). This means that the beam size is broadened more for longer wavelengths, and the sample is over-illuminated. Although this is a problem to be solved for applying MI–NR for small samples, it is not the main focus of this paper. Hence, we leave this issue for future work.

Finally, we performed time estimation for the measurement. The SOFIA reflectometer still has room for reducing the exposure time by a factor of 2 by increasing the beam power of the accelerator and by a factor of 2.5 by increasing the counting efficiency of the detector. Taking these reductions into consideration, the exposure time is estimated be less than 2 min by reference to the latest mirror for lower angles with a *Q*_*z*_ range from 0.08 to 0.56 nm^−1^, and less than 2 h is required for higher angles up to 2.6 nm^−1^ without taking the background into account. Furthermore, *operando* measurement with a time resolution of 10 min up to 2.6 nm^−1^ is possible by applying a carbon anode with an area of 35 × 35 mm, which is three times larger than that used in previous work (Kawaura *et al.*, 2016 ▸, 2020 ▸) but is a commercially available size.

## 4. Conclusion   

In this study, we have addressed the advantages of focusing optics with an elliptical supermirror for NR. In particular, for time-slicing measurements to investigate the kinetic processes of nanoscale interfaces during transient structural changes, focusing optics can be applied not only for improved time resolution owing to the higher flux on the sample but also to extend the accessible *Q*_*z*_ range for more detailed and reliable data analysis. Firstly, the performance of the focusing mirror that we have recently developed was presented from the perspective of the optical component for the SOFIA reflectometer at J-PARC MLF. It was confirmed that the minimum beam size at the sample position was 0.3 mm full width, which is less than half that of the existing Selene guide in the Amor reflectometer. Subsequently, a comparison was performed with the conventional optics, and the gain factor in the total flux was found to exhibit the maximum with a beam size of approximately 0.4 mm, which was slightly greater than the minimum beam size. Furthermore, the interfacial layer formed at the cathode of an LIB was successfully investigated with and without focusing optics, and the focusing optics succeeded in shortening the exposure time by a factor of 2.16 while obtaining consistent results with the conventional optics. Finally, a design for extending the *Q*_*z*_ range with focusing mirrors, that is, the optics for MI–NR in the SOFIA reflectometer, was proposed. The optics can be realized using a pair of rough focusing guides and two pairs of precise focusing mirrors with moderate and realistic design parameters, and can access the *Q*_*z*_ range from the total reflection to the typical upper limit for NR without any angle scan. The new optics are expected to be applicable to a small sample with a size of 15 × 15 mm as used in this study, and will be capable of *operando* measurements with a time resolution of 10 min up to *Q*_*z*_ = 2.6 nm^−1^ following further upgrades.

The focusing mirror system has already been installed on the SOFIA reflectometer and is ready for use. This is an important milestone for the practical utilization of focusing mirrors in NR. Moreover, the upgrade plan for MI–NR is in progress, and time-slicing measurements with improved time resolution using a wider *Q*_*z*_ range will hopefully be realized in the near future. We believe that this method offers the potential to open new doors for real-time interfacial nano­structure analysis, not only for electrode–electrolyte interfaces in batteries but also for various functional interfaces, such as catalyst–ionomer interfaces in fuel cells and emitter–injector interfaces in organic LEDs that are under operation.

## Supplementary Material

Supporting information file. DOI: 10.1107/S1600576720013059/ge5082sup1.pdf

## Figures and Tables

**Table:** Focusing mirror optics.

Layer	Thickness (nm)	Roughness (nm)	SLD (10^−4^ nm^−2^)
Substrate	–	–	3.52 (fixed)
SrRuO_3_	54.00 ± 0.06	3.29 ± 0.03	5.21 (fixed)
LiCoO_2_	25.24 ± 0.04	3.05 ± 0.03	3.83 (fixed)
Interfacial layer	12.56 ± 0.07	1.179 ± 0.008	5.239 ± 0.006
Electrolyte	–	1.54 ± 0.05	5.54 (fixed)

**Table:** Double-slit collimation.

Layer	Thickness (nm)	Roughness (nm)	SLD (10^−4^ nm^−2^)
Substrate	–	–	3.52 (fixed)
SrRuO_3_	53.96 ± 0.06	3.17 ± 0.03	5.21 (fixed)
LiCoO_2_	25.23 ± 0.04	3.02 ± 0.03	3.83 (fixed)
Interfacial layer	12.68 ± 0.08	1.114 ± 0.009	5.234 ± 0.007
Electrolyte	–	1.68 ± 0.07	5.54 (fixed)

**Table 2:** Design parameters of focusing mirror optics for MI–NR on the SOFIA reflectometer

Mirror	Focusing guide	Fine-focusing mirror
Focal length	4500 mm	2150 mm
Mirror length	1300 mm	300 mm
Axis length ratio	1000:6.8	1000:8.0
Critical angle	*m* = 2.5	*m* = 3
Cut-off wavelength	0.18 nm	0.18 nm

**Table 3:** *Q*_*z*_ values estimated with design parameters for MI–NR on the SOFIA reflectometer The minimum, efficient and highest *Q*_*z*_ values were evaluated with wavelengths of the maximum λ, 0.25 nm at the Maxwell peak and 0.18 nm at the cut-off wavelength of the focusing mirrors, respectively. The *Q*_*z*_ range factors for the repetition rates of 25 and 12.5 Hz with a single incident angle were 7.07 and 3.54, respectively.

Original angle	Offset angle	Minimum *Q*_*z*_	Efficient *Q*_*z*_	Highest *Q*_*z*_	*Q*_*z*_-range factor
1.04° (λ < 1.77 nm)	0°	0.13 nm^−1^	0.91 nm^−1^	1.26 nm^−1^	22.8 (= 2.95/0.13)
3.36° (λ < 0.88 nm)		0.83 nm^−1^	2.95 nm^−1^	4.08 nm^−1^	

1.04° (λ < 1.77 nm)	−0.1°	0.12 nm^−1^	0.82 nm^−1^	1.14 nm^−1^	24.5 (= 2.86/0.12)
3.36° (λ < 0.88 nm)		0.81 nm^−1^	2.86 nm^−1^	3.96 nm^−1^	

1.04° (λ < 1.77 nm)	−0.2°	0.10 nm^−1^	0.74 nm^−1^	1.02 nm^−1^	26.6 (= 2.77/0.10)
3.36° (λ < 0.88 nm)		0.78 nm^−1^	2.77 nm^−1^	3.84 nm^−1^	

1.04° (λ < 1.77 nm)	−0.3°	0.09 nm^−1^	0.65 nm^−1^	0.90 nm^−1^	29.2 (= 2.68/0.09)
3.36° (λ < 0.88 nm)		0.76 nm^−1^	2.68 nm^−1^	3.72 nm^−1^	

1.04° (λ < 1.77 nm)	−0.4°	0.08 nm^−1^	0.56 nm^−1^	0.78 nm^−1^	32.7 (= 2.60/0.08)
3.36° (λ < 0.88 nm)		0.73 nm^−1^	2.60 nm^−1^	3.60 nm^−1^	
