# Supplementary material for: Application of precise neutron focusing mirrors for neutron reflectometry: latest results and future prospects
Source: J Appl Crystallogr. 2020 Oct 26;53(Pt 6):1462–70. doi: 10.1107/S1600576720013059 (PMC7710489; doi:10.1107/S1600576720013059)
Supplement: Supplementary file 1 [file j-53-01462-sup1.pdf]

Supporting information

Application of Precise Neutron Focusing Mirror for Neutron Reflectometry – Latest Results and Future Prospects

Norifumi L. Yamada,[a,b]* Takuya Hosobata,[c] Fumiya Nemoto,[a,d] Koichiro Hori,[a,e] Masahiro Hino,[f] Jun Izumi,[g] Kota Suzuki,[g] Masaaki Hirayama,[g] Ryoji Kanno[h] and Yutaka Yamagata[c]

[a]Institute of Materials Structure Science, High Energy Accelerator Research Organization, Tokai, Naka, Ibaraki 319-1106, Japan, [b]Materials and Life Science Experimental Facility, Japan Proton Accelerator Research Complex, Tokai, Naka, Ibaraki 319-1195, Japan, [c]RIKEN Center for Advanced Photonics, RIKEN, Wako, Saitama 351-0198, Japan, [d]Department of Materials Science and Engineering, National Defense Academy, Yokosuka, Kanagawa 239-8686, Japan, [e]Sumitomo Rubber Industries, Ltd., Kobe, Hyogo 651-0071, Japan, [f]Kyoto University Institute for Integrated Radiation and Nuclear Science, Kumatori, Osaka 590-0494, Japan, [g]Interdisciplinary Graduate School of Science and Engineering, Tokyo Institute of Technology, Yokohama, Kanagawa 226-8502, Japan, and [h]All-Solid-State Battery Unit, Institute of Innovation Research, Tokyo Institute of Technology, Yokohama, Kanagawa 226-8503, Japan.
*Correspondence e-mail: norifumi.yamada@kek.jp

1. Slit condition for double slit optics

Figure S1 presents the beam paths of neutrons with double slit optics. For a sample with a length of l and an incident angle of neutrons, θ, the optics is required to make a beam with a size of $2l\tan\frac{\theta}{2}$ at the sample position. Then, the divergence of the beam $\Delta\theta$ is defined as the angle between the two paths illuminating the edges of the sample (red and orange lines in the figure). Here, the aperture of the slits and the beam size at the sample can be written with $\Delta\theta$ and the distance from the crossing point of the two paths as presented in the figure. These relations provided us the aperture of the slits for given θ, $\Delta\theta$, and l as follows:

$$S_1 = L_1\Delta\theta - l\theta,$$

$$S_2 = l\theta - L_2\Delta\theta,$$

where $\tan\theta \approx \theta$ and $\tan\Delta\theta \approx \Delta\theta$ can be applied as they are small for neutron reflectometry.

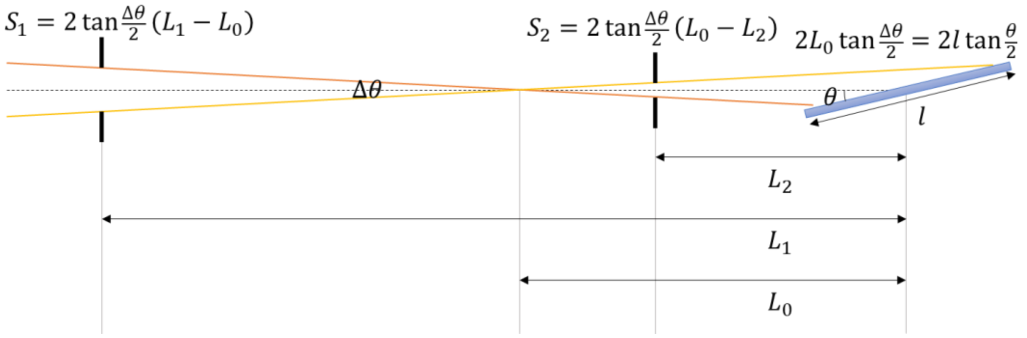

Figure S1 Beam paths of neutrons collimated by double slit optics.

Here, the product of the slit apertures is proportional to the beam flux

$$S_1 S_2 = -L_1 L_2 \left(\Delta\theta - \frac{L_1+L_2}{2L_1L_2} l\theta\right)^2 + \frac{(L_1-L_2)^2}{4L_1L_2}(l\theta)^2,$$

which can be maximized by choosing $\Delta\theta$ as

$$\Delta\theta = \Delta\theta_0 = \frac{L_1+L_2}{2L_1L_2} l\theta.$$

This condition gives a beam intensity profile with a triangle shape; when $\Delta\theta \neq \Delta\theta_0$, a trapezoid shape is obtained.

2. Slit condition for focusing mirror optics

Figure S2(a) presents how an image of a slit in a focal plane is projected by a focusing mirror on a sample position in the other focal plane. To simplify, the image reflected at a point on the mirror is considered in the figure (hereafter, $\tan\theta \approx \theta$ and $\tan\Delta\theta \approx \Delta\theta$ are applied in advance). On this point, neutrons are converged with an angular distribution of θ', and are reflected to the sample position with the same angular distribution. As the value of θ' is derived from the slit aperture and the distance from the point to the slit, the beam size at the sample can be evaluated with θ' and the distance from the point to the sample. With this relation, the aperture of the slit in the focal plane for given θ and l is described as

$$S_1 = \frac{L_1-L_3}{L_3} l\theta,$$

that is, the magnification factor becomes $\frac{L_3}{L_1-L_3}$.

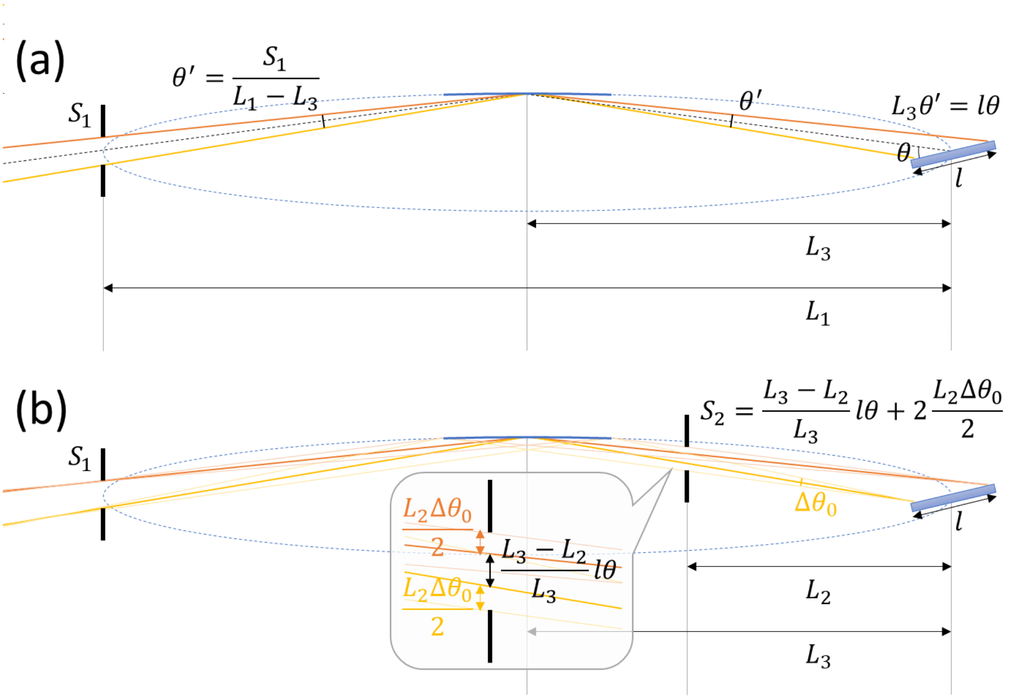

Figure S2 Beam paths of neutrons collimated by focusing mirror optics, where (a) is for explanation of magnification ratio and (b) is for the aperture at the second slit.

Next, we consider the beam size at the other slit, as presented in Fig. S2(b). The beam size reflected from the point can be evaluated with θ'. In addition, the beam divergence on the sample should be considered, in which the maximum beam divergence, $\Delta\theta_0$, is limited by the visual angle of the mirror from a point at the sample. The beam size at the slit is broadened by this effect and is converged at the sample position. Hence, the beam size at the slit can be the sum of these two terms, which is given as

$$S_2 = \frac{L_3 - L_2}{L_3} l\theta + L_2 \Delta\theta_0.$$

3. Specification of 5 μm slit

A slit with an aperture of 5 μm made of Cd was placed at the sample position and scanned to measure the intensity profiles because a beam size realized by our focusing mirror was too small to perform observations with a position-sensitive detector. The blades of the slit were manufactured with trapezoidal shapes by means of ultraprecision machining (Nagase-I, NPIC-M200) as the focusing mirror. Figure S1 shows the shape of the slit blade measured by an optical interferometer (ZYGO Newview7200). The short base at the top and leg angles of the slit were approximately 0.2 mm and 1° as designed, respectively. Then, the tops of the slit blades were allowed to contact each other, and the gap of the blades was adjusted to be 5 μm by checking the gap image using an optical microscope. Owing to the tapered shape, the transmission at the edge of the slit blade became negligible and could accept the beam divergence with 2° at maximum, which is larger than that of the focusing mirror, which is approximately 0.3°.

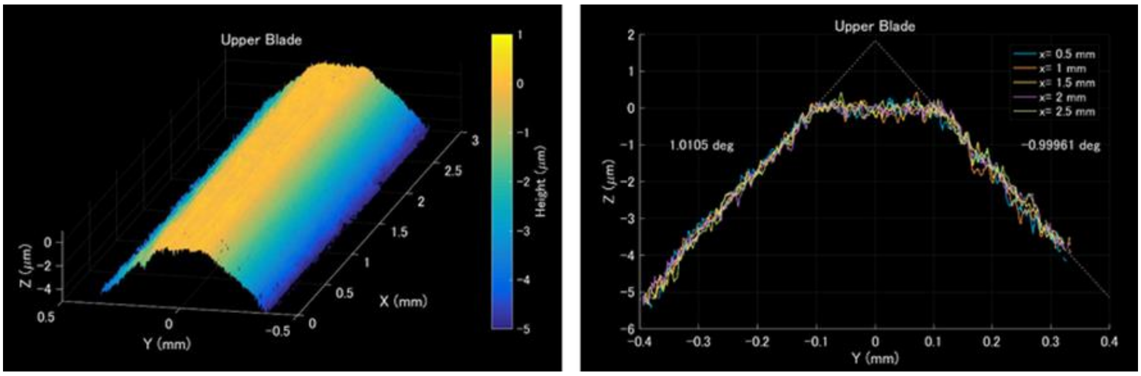

Figure S3 Shape of a Cd blade used for the 5 μm slit.

4. Effect of gravity for MI-NR

To evaluate the effect of gravity in the optics proposed for MI-NR, we performed ray-tracing considering the fall of neutrons by our own custom-made software. First, the analytical solution of the initial angle of a neutron at the virtual source was applied to hit neutrons at a point of the first mirror. Then, the angle and velocity of the reflected neutrons were evaluated as shown in Fig. S4, and the trajectory of the reflected neutrons was calculated using the values. When the trajectory crossed the surface of the second focusing mirror, we evaluated the crossing point numerically, modified the

angle and velocity of the reflected neutron based on the angle of the mirror at the point as the first mirror, and continued to calculate the trajectory till the sample position. If neutrons did not hit the surface of the second mirror, the trajectory calculation was continued without any modification.

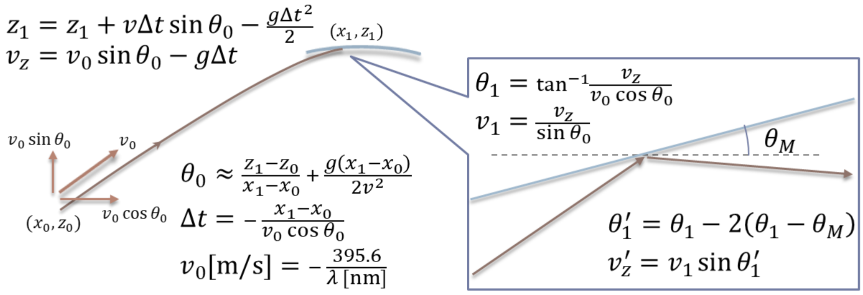

Figure S4 Treatment of reflection on ray-tracing considering the effect of gravity.

Figure S5 presents the results of the ray-tracing for different wavelengths. One is the thermal neutrons with the wavelength of 0.18 nm, and the other is the cold neutrons with the wavelength of 1.76 nm, which are the fastest and slowest neutrons available for the MI-NR optics, respectively. In the former case, the effect of gravity is negligible: all neutrons reflected by the first mirror hit the second mirror and converged at the sample position with a beam size of 2.4 μm. On the other hand, a part of the neutrons passed under the second mirror, and the reflected neutrons converged just short of the sample position in the case of slow neutrons. These are caused by the fall of neutrons, and the beam size at the sample position was evaluated to be 110 μm, which was not critical but was non-negligible to realize the smaller beam size for such slow neutrons with optics.

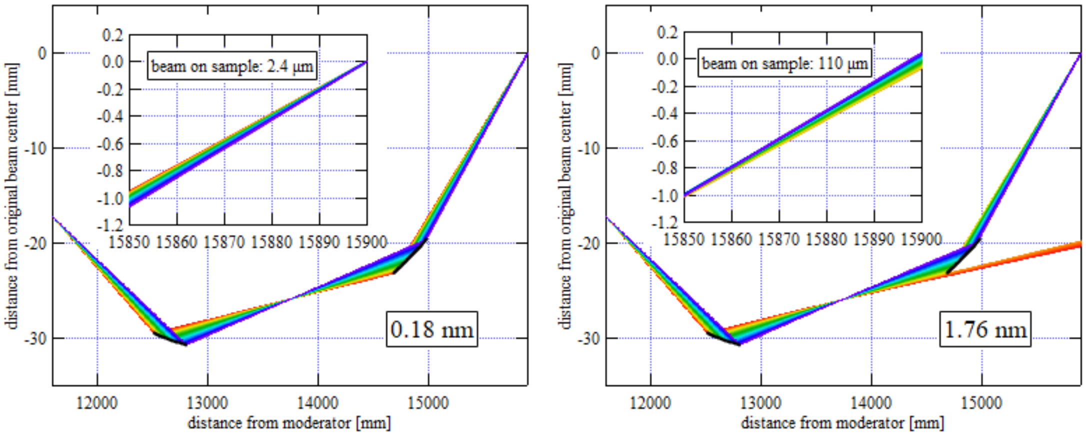

Figure S5 Results of ray-tracing for optics of MI-NR with thermal neutrons (0.18 nm) and cold neutrons (1.76 nm) considering the effect of gravity.
